# HK-2 cell response to TGF-β highly depends on cell culture medium formulations

**DOI:** 10.1007/s00418-023-02237-x

**Published:** 2023-09-26

**Authors:** Gantsetseg Garmaa, Anna Manzéger, Samaneh Haghighi, Gábor Kökény

**Affiliations:** 1 Institute of Translational Medicine, Semmelweis University, Nagyvárad tér 4, Budapest, 1089 Hungary; 2 International Nephrology Research and Training Center, Semmelweis University, Nagyvárad tér 4, Budapest, 1089 Hungary

**Keywords:** Kidney fibrosis, TGF-beta, EMT, Cell culture, PTEC, Transcription factor

## Abstract

The immortalized human renal proximal tubular epithelial cell line HK-2 is most commonly used to study renal cell physiology and human kidney diseases with tubulointerstitial fibrosis such as diabetic nephropathy, obstructive uropathy or allograft fibrosis. Epithelial-to-mesenchymal transition (EMT) is the main pathological process of tubulointerstitial fibrosis in vitro. Transforming growth factor-beta (TGF-β) is a key inducer of EMT. Several pro-fibrotic gene expression differences have been observed in a TGF-β-induced EMT model of HK-2 cells. However, growth conditions and medium formulations might greatly impact these differences. We investigated gene and protein expression of HK-2 cells cultured in six medium formulations. TGF-β1 increased the expression of *ACTA2*, *TGFB1, COL4A1, EGR2, VIM* and *CTGF* genes while reducing *PPARG* in all medium formulations. Interestingly, TGF-β1 treatment either increased or decreased *EGR1*, *FN*, *IL6* and *C3* gene expression, depending on medium formulations. The cell morphology was slightly affected, but immunoblots revealed TGFB1 and vimentin protein overexpression in all media. However, fibronectin expression as well as the nuclear translocation of EGR1 was medium dependent. In conclusion, our study demonstrates that, using the HK-2 in vitro model of EMT, the meticulous selection of appropriate cell culture medium formulation is essential to achieve reliable scientific results.

**Supplementary Information:**

The online version contains supplementary material available at 10.1007/s00418-023-02237-x.

## Introduction

The kidney is a complex organ composed of at least 16 functionally distinct epithelial cell types (Balzer et al. 2022). The proximal tubular epithelial cells (PTEC), along with the tubular vasculature, make up > 80% of the renal cortex and are among the most abundant epithelial cell types (Balzer et al. 2022). They play essential roles in fluid, amino acid and sodium reabsorption and contribute significantly to pathological changes within the cortical tubulointerstitium (Sato et al. 2020).

To study the function of PTEC cells, specialized isolation and culture techniques were developed that eliminate interference from other renal cell types, hemodynamics and neural activity. Several human cell lines, such as ciPTEC (Wilmer et al. 2010), RPTEC/TERT1 (Wieser et al. 2008) and SA7K (Li et al. 2017), have been newly established over the last decade. Still, the immortalized proximal tubule epithelial cell line HK-2 is most commonly used (Valdés et al. 2021) to study cell physiology, pharmacology (Jenkinson et al. 2012) and toxicology (Li et al. 2017) as well as various kidney diseases. HK-2 was derived from normal adult human renal cortex and immortalized through transduction with human papillomavirus (HPV16) E6/E7 genes, maintained for up to 30 passages (Ryan et al. 1994).

Regardless of the lack of some transporters, HK-2 is the mainly used in vitro model to study renal epithelial-to-mesenchymal transition (EMT) (Wang et al. 2018). EMT is recognized as an essential component of tissue fibrogenesis following kidney injury (Wang et al. 2018). Several studies show that tubular epithelial cells undergo phenotypic conversion in vitro after being incubated with fibrogenic transforming growth factor beta-1 (TGF-β1) (Tian and Phillips 2003). During EMT, the cells lose their epithelial proteins, such as E-cadherin and zonula occludens-1 (ZO-1), and start to produce the mesenchymal markers vimentin, α-SMA, type I collagen, fibronectin (Liu 2004) and connective tissue growth factor (CTGF) (Cheng et al. 2006).

Although HK-2 cell providers recommended the use of serum-free medium supplemented with bovine pituitary extract (BPE) and human recombinant epidermal growth factor (rEGF) (Ryan et al. 1994), most of the biomedical studies documented the use of DMEM or DMEM/F12 media supplemented with fetal bovine serum (FBS) or hormones (Bozic et al. 2011, 2020; Kang et al. 2019). Thus, we aimed to establish and compare the TGF-β1-induced EMT model in HK-2 cells in various culture medium formulations commonly used for epithelial cells. We further aimed to investigate how different culture media affect the pro-fibrotic gene expression pattern.

Here, we report different cell behavior (growth rate, morphology, differential gene and protein expression) upon changing the medium culture formulations. Our results underline that appropriate cell culture media should be carefully selected for specific scientific questions to avoid potential misinterpretation of experimental results obtained from the widely used HK-2 in vitro model.

## Materials and methods

### Cell culture

HK-2 cells were purchased from the American Type Cell Collection (ATCC, #CRL-2190). Cells were cultured in T75 flasks with six different growth media (Table 1) at 37 °C in a humid atmosphere of 95% air and 5% CO_2_. The medium was refreshed every 48 to 72 h until the desired confluency. After 3 to 5 days, when cells reached 70–80% confluence, HK-2 cells were trypsinized and passaged at a 1:3 ratio or seeded on 6 or 24-well plates at the desired density for immediate experimental use. HK-2 cells were seeded ∼10^5^ cells or 3 × 10^4^ per well in 6- or 24-well plates, respectively, and cultured in different growth media overnight, then serum starved for 24 h. Recombinant human TGF-β1 (10 ng/ml, Sigma-Aldrich) was next added to the appropriate wells for 24 h. Microphotographs of treated and untreated cells in the culture plate wells were taken with Nikon Coolpix 4500 digital camera attached to a Nikon Eclipse TS100 microscope at 200 × magnification. Cells were then harvested in Trizol (Invitrogen, Thermo, USA) for total RNA isolation according to the manufacturer’s protocol or in ice-cold RIPA buffer for protein extraction, as mentioned below.

**Table 1:** Cell culture media and their formulation

Medium abbreviations	Formulation
DMEM 2%	DMEM with 1000 mg/l glucose, l-glutamine, sodium bicarbonate (Invitrogen, Carlsbad, CA), and 2% fetal bovine serum (Invitrogen), 50 U/ml penicillin and 50 μg/ml streptomycin (Invitrogen)
DMEM 5%	DMEM with 1000 mg/l glucose, l-glutamine, sodium bicarbonate (Invitrogen, Carlsbad, CA) and 5% fetal bovine serum (Invitrogen), 50 U/ml penicillin and 50 μg/ml streptomycin (Invitrogen)
DMEM 10%	DMEM with 1000 mg/l glucose, l-glutamine, sodium bicarbonate (Invitrogen, Carlsbad, CA) and 10% fetal bovine serum (Invitrogen), 50 U/ml penicillin and 50 μg/ml streptomycin (Invitrogen)
DMEM/F12 10%	DMEM/F12 medium contains a 1:1 mixture of DMEM medium and Ham′s F12 medium, with l-glutamine, 15 mM HEPES, sodium bicarbonate (Invitrogen, Carlsbad, CA) and 10% fetal bovine serum (Invitrogen), 50 U/ml penicillin and 50 μg/ml streptomycin (Invitrogen)
PTEC (hormonally defined)	DMEM/F12 (Invitrogen, Carlsbad, CA) supplemented with 2% fetal bovine serum (Invitrogen), 5 μg/ml insulin, 5 μg/ml transferrin, 5 ng/ml selenium, 40 ng/ml hydrocortisone, 5 pg/ml triiodo-1-thyronine, 50 U/ml penicillin and 50 μg/ml streptomycin (Invitrogen)
KSFM	Keratinocyte serum-free growth medium (Invitrogen, Carlsbad, CA) supplemented with 0.05 mg/ml bovine pituitary extract (BPE), 5 ng/ml human recombinant epidermal growth factor (rEGF) (Invitrogen, Carlsbad, CA), 50 U/ml penicillin and 50 μg/ml streptomycin (Invitrogen)

*BPE* Bovine pituitary extract, *rEGF* human recombinant epidermal growth factor, *DMEM 2%* DMEM with 2% FBS supplemented medium, *DMEM 5%* DMEM with 10% FBS supplemented medium, *DMEM/F12 10%* DMEM with 10% FBS supplemented medium, *PTEC* hormonally defined medium, *KFSM* keratinocyte serum-free growth medium

### RNA isolation and qPCR

Total RNA from Trizol samples was extracted according to the manufacturer’s protocol. RNA concentration and purity were verified on a Nanodrop 2000 (Thermo, USA) and then reverse transcribed using the High-Capacity cDNA kit (Applied Biosystems/life Technologies, Carlsbad, CA, USA), as stated previously (Kökény et al. 2022). Each PCR reaction with specific primers (Table 2) was performed on a Bio-Rad CFX thermal cycler (Bio-Rad Hungary, Budapest, Hungary) in duplicates using the SensiFast SYBR Green PCR Master Mix (Bioline, Germany). The specificity and effectivity of PCR reactions were verified by melting curve analysis. Target gene expression was normalized to glyceraldehyde-3-phosphate dehydrogenase (GAPDH) mRNA expression using the 2^−ΔΔCt^ formula and expressed as fold expression relative to a control sample. Each gene expression is presented as mean ± standard deviation (SD).

**Table 2:** Primer sequences (5′–3′) used for quantitative PCR

*hACTA2*, forward	5′-AGATCAAGATCATTGCCC-3′
*hACTA2*, reverse	5′-TTCATCGTATTCCTGTTTGC-3′
*hCOL4A1*, forward	5′-AAAGGGAGATCAAGGGATAG-3′
*hCOL4A1*, reverse	5′-TCACCTTTTTCTCCAGGTAG-3′
*hC*3, forward	5′-CGGATCTTCACCGTTCAACCA-3′
*hC3*, reverse	5′-GATGCCTTCCGGGTTCTCAA-3′
*hCTGF*, forward	5′-TTAAGAAGGGGCAAAAAGTGC-3′
*hCTGF*, reverse	5′-CATACTCCACAGAATTAGCTC-3′
*hEGR1*, forward	5′-ACCTGACCGCAGAGTCTTTT-3′
*hEGR1*, reverse	5′- GAGTGGTTTGGCTGGGGTAA -3′
*hFN*, forward	5′-CCATAGCTGAGAAGTGTTTTG-3′
*hFN*, reverse	5′-CAAGTACAATCTACCATCATCC-3′
*hGAPDH*, forward	5′-CATGAGAAGTATGACAACAGCCT-3′
*hGAPDH*, reverse	5′-AGTCCTTCCACGATACCAAAGT-3′
*hIL6*, forward	5′-GCAGAAAAAGGCAAAGAATC-3′
*hIL6*, reverse	5′-CTACATTTGCCGAAGAGC-3′
*hTGFB1*, forward	5′-GGA AAT TGA GGG CTT TCG CC-3′
*hTGFB1*, reverse	5′-CCG GTA GTG AACCCG TTG AT-3′
*hVIM*, forward	5′-GGAAACTAATCTGGATTCACTC-3′
*hVIM*, reverse	5′-CATCTCTAGTTTCAACCGTC-3′
*hPPARG*, forward	5′- AAAGAAGCCAACACTAAACC -3′
*hPPARG*, reverse	5′- TGGTCATTTCGTTAAAGGC -3′

### Immunoblot

HK-2 cells were lysed in ice-cold RIPA buffer containing “Complete Mini” protease inhibitor cocktail (Roche, Mannheim, Germany). Protein concentration was determined using BCA assay (Thermo Scientific, Waltham, MA, USA). Equal amounts (20 μg) of protein were loaded in Laemmli buffer on 8% or 12% SDS-polyacrylamide gels. Separated samples were transferred to nitrocellulose membranes, blocked with 5% skim milk and incubated with primary antibodies (1:2000, see Supplemental Table 1) overnight at 4 °C and then with the appropriate HRP-conjugated secondary antibodies as described previously (Kökény et al. 2022). Blots were visualized with enhanced chemiluminescence (ECL) detection kit (Thermo).

### Immunofluorescence

The protein expressions of EGR1 and TGFB1 were assessed using immunofluorescence. HK-2 cells (10,000/well) were seeded on glass coverslips in a 24-well plate. After 24-h treatment with 10 ng/ml TGF-β1 in serum-free medium, cells were washed and fixed in methanol, then permeabilized with 0.25% Triton-X. Non-specific binding of secondary antibody was blocked using 2% donkey serum for 30 min. EGR1 (1:200) and TGF-β (1:200) antibodies (see Supplemental Table 1) or PBS for negative controls (omitting primary antibody) was applied overnight, and then the cells were incubated with donkey anti-rabbit IgG-A594 (1:200) (Jackson Immunoresearch) at room temperature in the dark. Coverslips were mounted with Vectashield containing DAPI (Vector Laboratories, Burlingame, CA, USA). Cells were visualized and photographed under UV light using a Leica DMR-HC microscope at 400 × magnification. Primary antibody specificity was evaluated on immunoblots.

### Statistical analysis

Experimental data are presented as mean ± SD. Statistical analysis was performed using SPSS 28.0.0 for Windows (SPSS Inc). Shapiro-Wilk test was performed to analyze normal distribution of the data. Pairwise comparisons were conducted using the independent samples Mann-Whitney *U* test or Kruskal-Wallis test, followed by Bonferroni correction, as indicated, to analyze normalized expression levels. Spearmen and Pearson’s correlation was used for the relationship between continuous and ordinal variables. The significance level was *p* < 0.05 at the 95% confidence level.

## Results

### Cell morphology

**Fig. 1:**
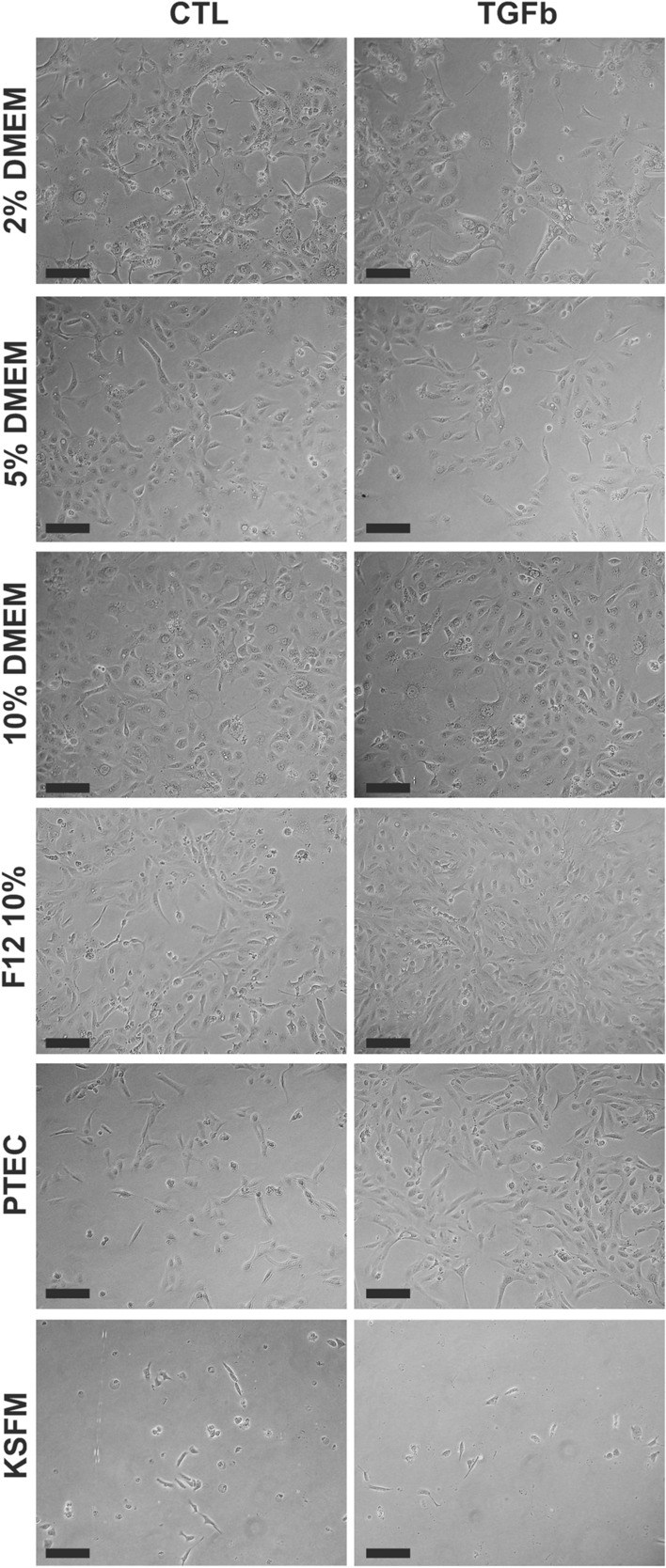
Effect of different medium formulations on HK-2 cell morphology in control (CTL) and TGF-β1-treated (TGFb) groups. Light microscopy, 200 × magnification; scale bar represents 100 μm. *2% DMEM* DMEM with 2% FBS supplemented medium, *5% DMEM* DMEM with 5% FBS supplemented medium, *10% DMEM* DMEM with 10% FBS supplemented medium, *F12 10%* DMEM/F12 with 10% FBS supplemented medium, *PTEC* hormonally defined medium, *KFSM* keratinocyte serum-free growth medium

We examined the effect of six culture medium formulations (see Table 1) on cell morphology. Cells without TGF-β1 treatment depicted epithelial morphology, whereas TGF-β1 treatment (10 ng/ml) for 24-h induced a slightly elongated morphology in all medium formulations (Fig. 1). However, HK-2 cultured in KFSM appeared smaller and did not replicate as fast as cells cultured in DMEM or DMEM F12-based formulations on day 5 after the passage. At sub-confluent density, HK-2 in DMEM 5% FBS most likely appeared round-shaped and polygonal (Fig. 1). HK-2 cells reached 80% confluence at culture day 7 in KFSM and day 5 in DMEM and DMEM F12-based media. On day 7, space was still visible in a T-75 flask with KFSM and DMEM 2% FBS medium. In addition, cells in DMEM with 10% FBS appeared larger (Fig. 1).

### Gene and protein expression variations related to EMT and pro-fibrotic markers

**Fig. 2:**
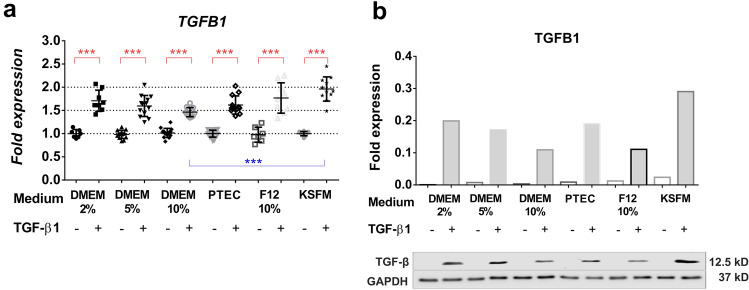
TGF-β1 mRNA *(TGFB1)* and protein (TGFB1) expression of HK-2 cells in different medium formulations. **a** Representative qPCR results of TGF-β1-induced effects on HK-2 cells cultured in different medium formulations for 24 h. The gene expression of *TGFB1* was normalized to *GAPDH* and indicated as fold expression relative to the respective controls (mean ± SD). Significant differences between the control vs. TGFb (*n* = 7–16/group) group shown as a red line and TGFb vs. TGFb groups between culture media shown as blue line are indicated as *p* < 0.05*, *p* < 0.01** and *p* < 0.001***. Independent samples Mann-Whitney *U* test and Kruskal-Wallis test. **b** Representative immunoblot of TGFB protein expression is shown for each group; GAPDH was used as a loading control. *TGFB1*: transforming growth factor mRNA, *TGFB* transforming growth factor beta protein, *CTL* control group, *TGFb* transforming growth factor beta-1 (10 ng/ml)-treated group

**Fig. 3:**
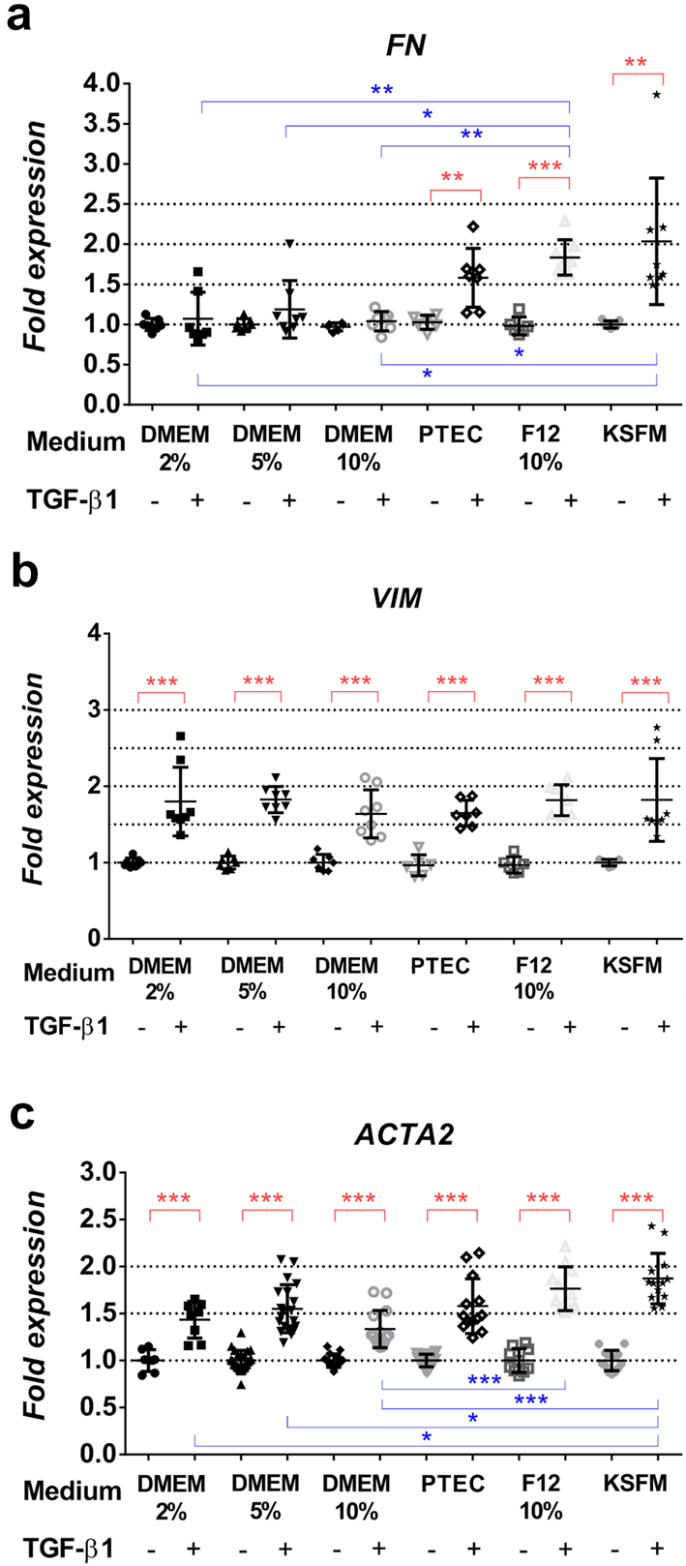
EMT-related gene expression pattern of HK-2 cells. Gene expression of control (CTL) and TGF-β1-treated (TGFb) cells in each culture medium is expressed as fold expression relative to the corresponding controls. Expression of each gene was normalized to GAPDH, and mean expression levels with standard deviation (± SD) are shown; **a** *FN* (*n* = 7–8/group), **b** *VIM* (*n* = 7–8/group), **c** *ACTA2* (n-7–24/group). Significant differences within the groups are indicated as red lines with *p* < 0.05*, *p* < 0.01** and *p* < 0.001***. Inter-group differences are blue lines with *p* < 0.05*, *p* < 0.01** and *p* < 0.001***. Independent samples Mann-Whitney *U* test and Kruskal-Wallis test

TGF-β1 promoted phenotype changes of HK-2 cells toward EMT, as confirmed by the slightly elongated morphology (Fig. 1), the significantly increased TGFB1 gene (Fig. 2a) and protein expression (Fig. 2b), and highly expressed mesenchymal markers by qPCR (Fig. 3a–c). EMT-related changes at the mRNA level were observed regardless of the culture medium formulations (Fig. 3). The mRNA expression of vimentin *(VIM)* was induced in all formulations (Fig. 3b); however, immunoblot showed its overexpression in all media except DMEM 5% (Supplementary Fig. S1a, c). TGF-β1 partially induced the mRNA expression of *FN* in PTEC 2%, DMEM F12 10% and KFSM (Fig. 3a) and the protein expression in almost all media except DMEM/F12 10% and KFSM (Supplementary Fig. S1b, c).

**Fig. 4:**
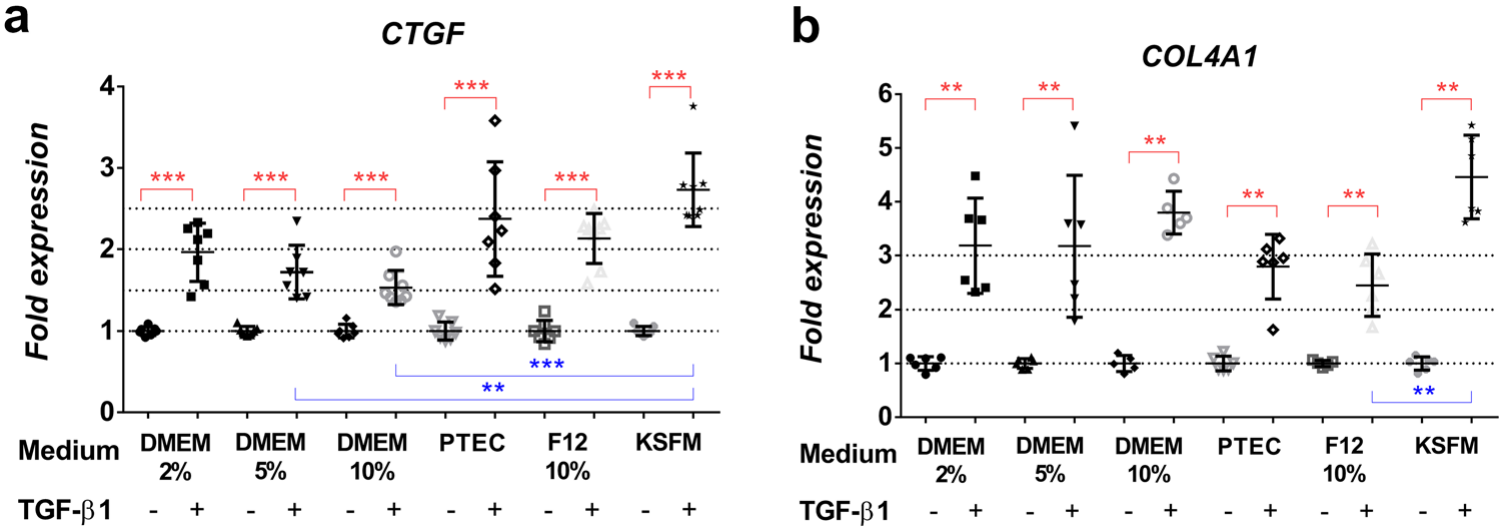
Pro-fibrotic gene expression pattern depends on culture medium formulation. Gene expression of control (CTL) and TGF-β1-treated (TGFb) HK-2 cells in each culture medium are expressed as fold expression relative to the corresponding controls as follows: **a** CTGF (*n* = 7–8/group), **b** *COL4A1* (*n* = 5–6/group). Expression of each gene was normalized to GAPDH and shown as mean ± SD. Significant differences within the groups are indicated as red lines with *p < 0.05, **p < 0.01 and ***p < 0.001. Inter-group differences are blue lines with *p < 0.05, **p < 0.01 and ***p < 0.001. Independent samples Mann-Whitney *U* test and Kruskal-Wallis test

**Fig. 5:**
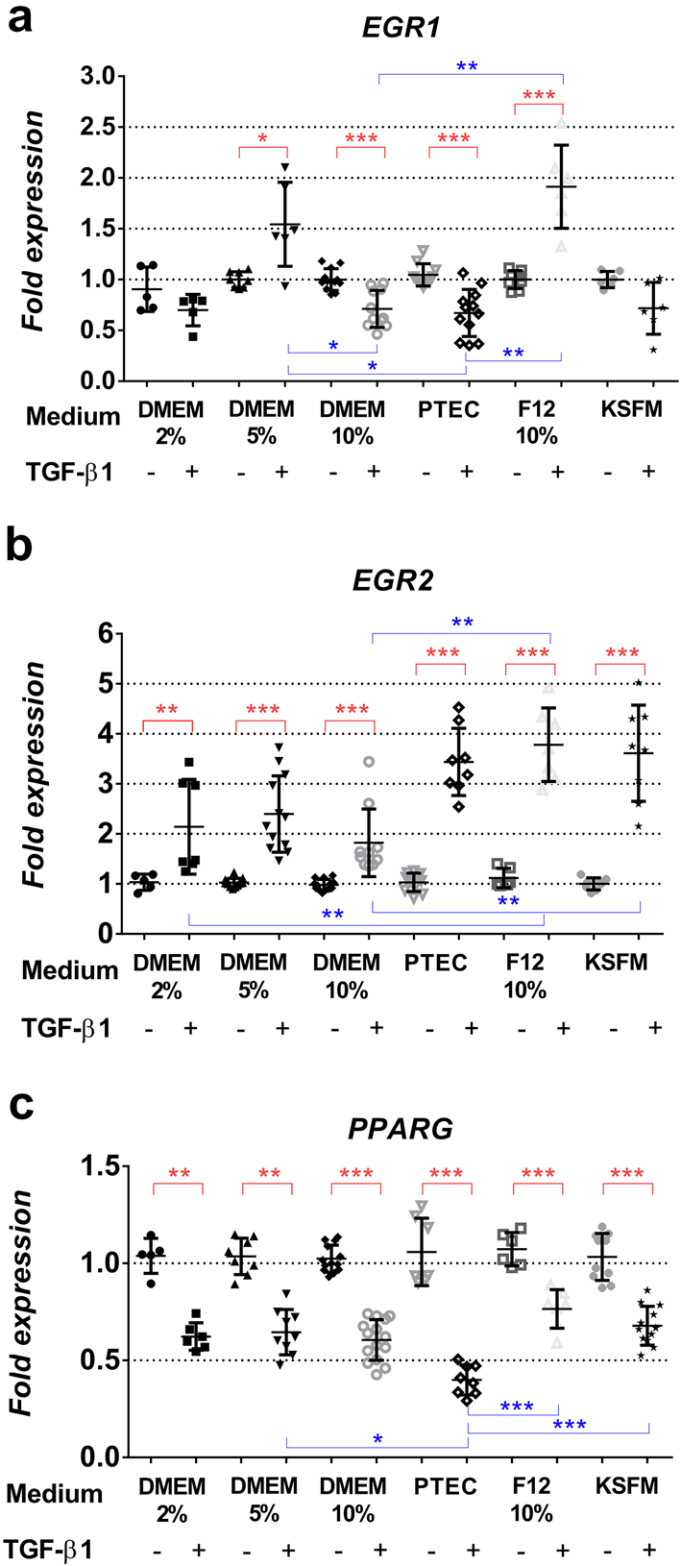
Gene expression pattern of transcription factors upon culture medium formulations. Gene expression of control (CTL) and TGF-β1-treated (TGFb) HK-2 cells in each culture medium are expressed as fold expression relative to the corresponding controls as follows: **a** *EGR1* (*n* = 5–12/group), **b** *EGR2* (*n* = 5–14/group), **c** *PPARG* (*n* = 5–13/group). Expression of each gene was normalized to GAPDH and shown as mean ± SD. Significant differences within the groups are indicated as red lines with *p < 0.05, **p < 0.01 and ***p < 0.001. Inter-group differences are blue lines with *p < 0.05, **p < 0.01 and ***p < 0.001. Independent samples Mann-Whitney *U* test and Kruskal-Wallis test

*CTGF, COL4A1* and *EGR2* expressions were significantly increased upon TGF-β1 treatment in all investigated media (Fig. 4a, b, Fig. 5b). Interestingly, medium formulations had a substantial impact on pro-fibrotic *EGR1* expression. TGF-β1 treatment increased *EGR1* mRNA expression significantly only in DMEM 5% and DMEM/F12 10% media, but in PTEC 2% and DMEM 10%, *EGR1* decreased (Fig. 5a, Table 3).

**Table 3:**
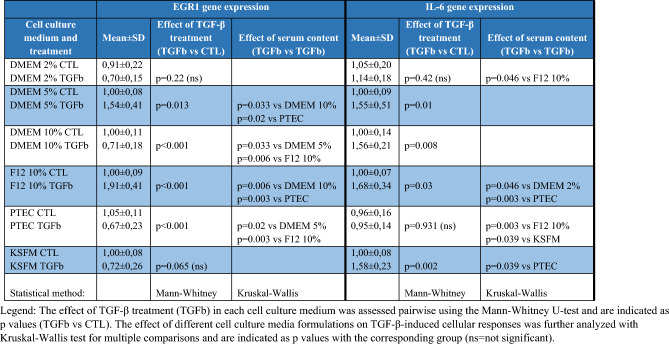
TGF-β-induced pro-fibrotic EGR1 and inflammatory IL-6 gene expression responses based on cell culture media

*PPARG* expression decreased significantly in all culture media upon TGF-β1 treatment (Fig. 5c), although there were differences between culture media; the most potent effect was observed using PTEC formulation (0.39 ± 0.08 in PTEC 2% vs. 0.68 ± 0.10 in KFSM and 0.76 ± 0.09 in DMEM F12 10%).

**Fig. 6:**
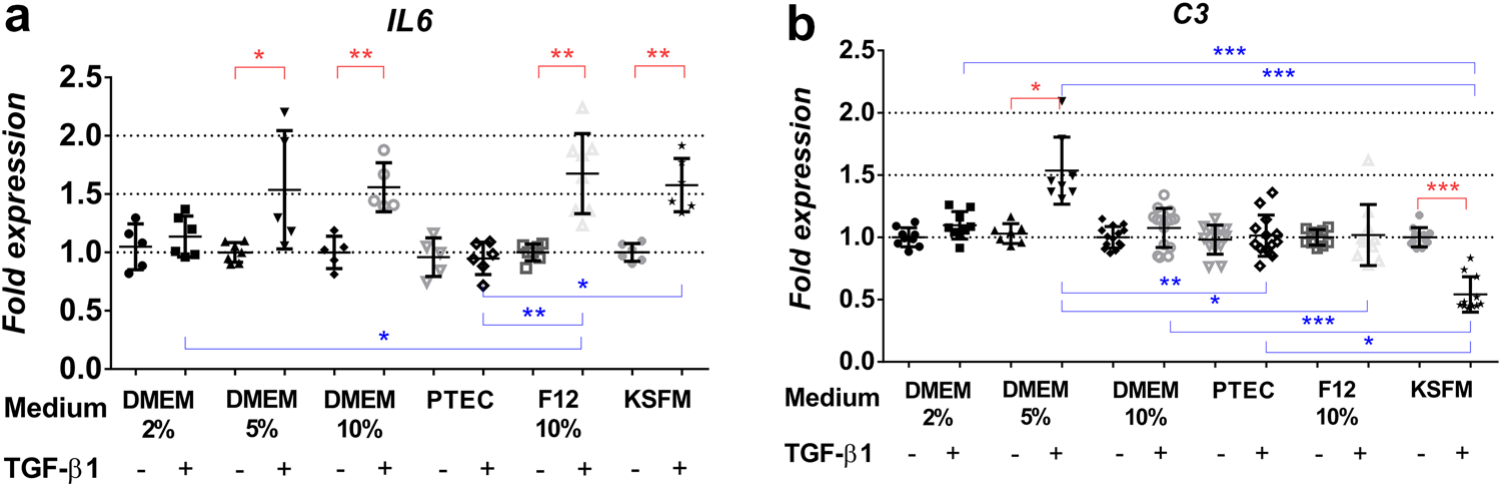
Cell culture medium-dependent expression of inflammatory genes. Gene expression of control (CTL) and TGF-β1-treated (TGFb) HK-2 cells in each culture medium are expressed as fold expression relative to the corresponding controls as follows: **a** *IL*6 (*n* = 5–7), **b** *C3* (*n* = 7–11/group). Gene expression was normalized to GAPDH and shown as mean ± SD. Significant differences within the groups are indicated as red lines with *p ≤ 0.05, **p ≤ 0.01 and ***p ≤ 0.001. Inter-group differences are blue lines with *p ≤ 0.05, **p ≤ 0.01 and ***p ≤ 0.001. Independent samples Mann-Whitney *U* test and Kruskal-Wallis test

Among the inflammatory markers, *IL6* mRNA was not induced in TGF-β1-treated HK-2 cells cultured in PTEC 2% medium in addition to the DMEM 2% (Fig. 6a, Table 3). The expression of the complement C3 protein-coding gene, C3, decreased in KSFM and increased in DMEM 5% but did not change in any other media upon TGFβ1 treatment (Fig. 6b).

### Effect of serum concentration and additive hormones on the EMT process

To clarify the effect of serum concentration in a culture medium, we cultured HK-2 cells in DMEM supplemented with 2%, 5% or 10% FBS. By Spearman’s correlation, there was no significant correlation between the percentage of FBS and the expression of mRNAs listed in Figs. 3, 4, 5 and 6 in the abovementioned three media.

To further clarify the effect of different formulations besides FBS, we cultured HK-2 cells on a hormonally defined medium (PTEC, Table 2). Notably, immunoblot depicted significantly (1.7-fold) overexpressed fibronectin in PTEC medium (Supplemental Fig. S1b).

We also compared DMEM vs. DMEM F12-based medium supplemented with 10% FBS. The mean mRNA expression of *FN, ACTA2, EGR1* and *EGR2* was differentially expressed in TGF-β1-induced HK-2 cells cultured in these two media (*p* < 0.05) (Figs. 3a, c, 5a, b). Interestingly, FN protein expression was reduced by 0.73-fold in DMEM F12 10% (Supplementary Fig. S1b).

Finally, these data indicated that TGF-β1 stimulates partial EMT and pro-fibrotic program of HK-2 cells differently depending on cell culture medium formulations.

### Different cellular localization of EGR1 protein by immunocytochemistry

**Fig. 7:**
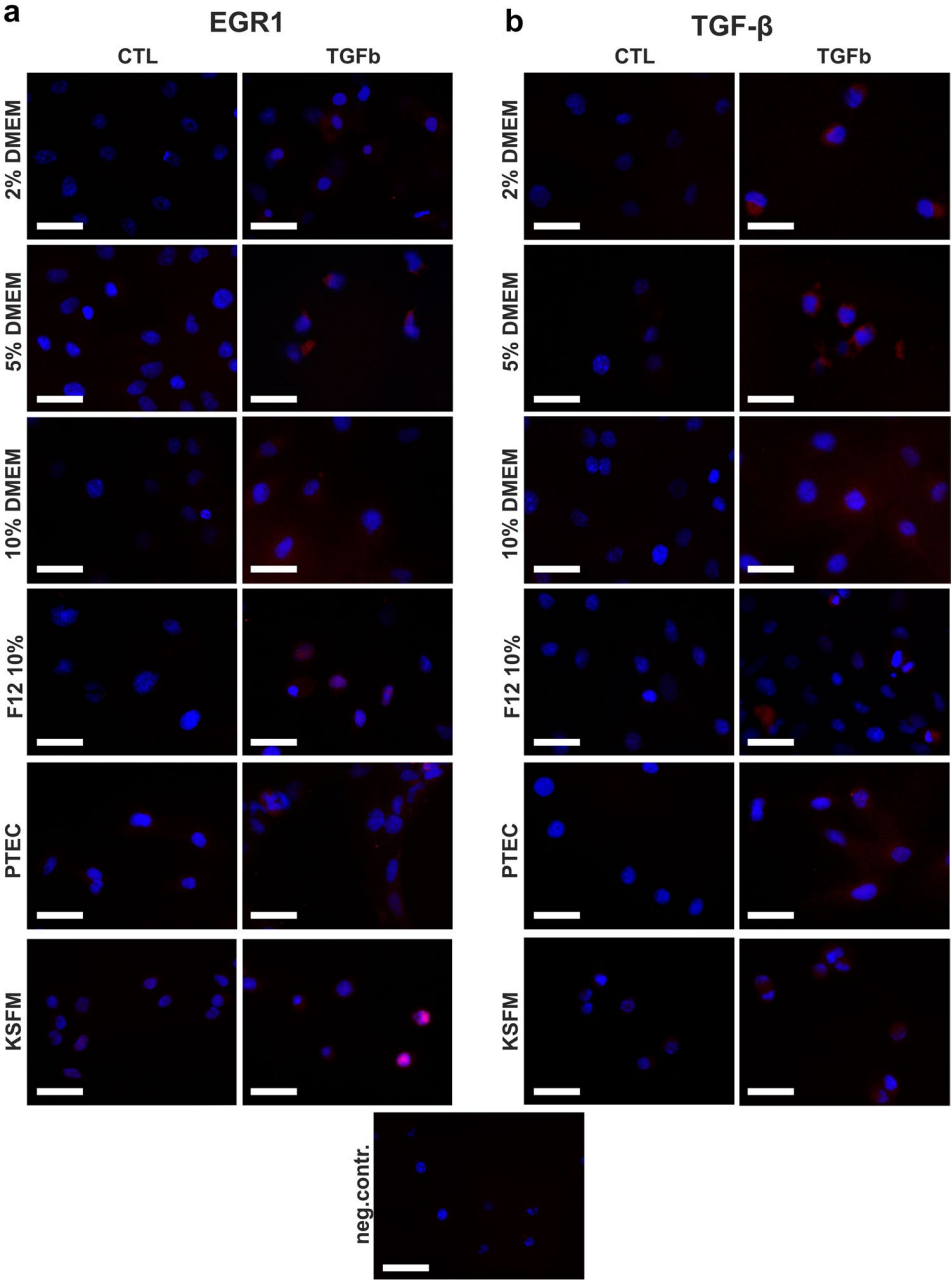
EGR1 and TGFB1 immunocytochemistry of TGF-β1 induced HK-2 cells. EGR1 (**a**) and TGFB1 (**b**) protein expression of control and TGF-β1-treated (10 ng/ml for 24 h) cells in different medium formulations. Immunofluorescence, 400 × magnification. Neg. contr.: representative photograph of the negative control sample (omitting the primary antibodies). Scale bar represents 50 μm. Red stain: EGR1 or TGFB1; blue stain: nuclear stain (DAPI). Magenta: double stain

Based on the differential expression of mRNAs in culture medium formulations, we investigated the expression pattern of EGR1 in TGF-β-induced HK-2 cells by immunofluorescence. TGF-β induced EGR1 translocation to the nucleus in DMEM F12 10% and KFSM media indicating transcriptional activation (Fig. 7a). Cells grown in other media (DMEM 2%, 5%, 10% and PTEC 2%) depicted only cytoplasmic overexpression of EGR1 (Fig. 7a). Still, according to both gene expression and immunoblot analysis, we observed TGFB1 protein expression induced by TGF-β1 treatment independent of the used culture medium (Fig. 7b).

## Discussion

HK-2 is a widely used in vitro model to study EMT in kidney tubules. However, published studies use different cell culture media (Bozic et al. 2011, 2020; Li et al. 2005), which may lead to inhomogeneous or even controversial experimental results. Thus, we investigated how the most commonly used culture medium formulations affect cell behavior upon TGF-β1 administration, depicted as cell morphology, gene and protein expression pattern. To the best of our knowledge, this is the first comparative study on HK-2 cell morphology and expression pattern in different culture medium formulations.

### Cell morphology

In general, TGF-β1 induced EMT in all used culture media. Our results indicate that HK-2 cells cultured in serum-free and DMEM 2% medium showed a slow growth rate and smaller cell size than those cultured in other media. Based on our study, we found that cells grown in a higher proportion of FBS (10%) appeared enlarged. However, no evidence suggests a connection between PTEC cell size and FBS concentration. Interestingly, one study reported an increase in size, similar to human pancreatic cancer spheres (Sasaki et al. 2019), when FBS was present. Further research is necessary to fully understand the effects of FBS on cell growth and size. In contrast, cells grown in a hormonally defined medium looked more flattened and elongated. Notably, the slower growth rate of cells in the DMEM 2% medium was predictable as FBS is the main component of culture media supporting cell growth (Yao and Asayama 2016; Puck et al. 1958).

### Gene and protein expression

Despite the morphological differences, gene expression of the classic EMT markers *ACTA2, TGFB, VIM* and *CTGF* was significantly upregulated in TGF-β1-induced HK-2 cells regardless of the culture medium, indicating that any of these medium formulations is feasible for investigation of the expression changes of these genes in a TGF-β1-induced system. This corroborates previously published data (Brennan et al. 2012). Our EMT model partially induced the expression of mesenchymal (α-SMA, vimentin, fibronectin) markers. Notably, protein expression differed somewhat from the corresponding mRNA expression results. Khundmiri and colleagues noted that protein expression in HK-2 cells could differ from mRNA expression seen in human kidney and primary PTEC because of the low percentage (26%) of proximal tubule-specific transcripts detected in the HK-2 cell line, as obtained from RNA-Seq (Khundmiri et al. 2021).

In addition, the observed differential gene and protein expression could be due to a lack of serum in the serum-free medium or interactions with other additives in the culture medium. Regarding specific additives, EGF is considered a mitogen and supports cell proliferation, but its combination with TGF-β has been reported to cause primary PTEC cell hypertrophy and excessive accumulation of extracellular matrix proteins (Franch et al. 1995). This may explain the higher expression of *COL4A1* in the KFSM medium supplemented with EGF compared to other media in our study.

Peroxisome proliferator receptor-gamma (PPAR-γ) is a nuclear receptor superfamily member and has been demonstrated to ameliorate renal fibrogenesis (Zhao et al. 2016; Németh et al. 2019)*. PPARG* is downregulated in fibrosis, and PPAR-γ agonists are promising therapeutic agents in fibrotic diseases (Kökény et al. 2020; Ghallab and Seddek 2020; Németh et al. 2019). Our results corroborate these studies depicting a strong TGF-β1-related reduction of *PPARG* expression in all investigated medium formulations (Fig. 5c).

Early growth response factor-1 and -2 (EGR1, EGR2) have been implicated as important pro-fibrotic transcription factors in kidney diseases (Bhattacharyya et al. 2013, 2011; Ho et al. 2016; Sun et al. 2014; Vollmann et al. 2017). We have previously demonstrated a PPARG-EGR1 axis in TGF-β-induced renal fibrosis (Németh et al. 2019). Interestingly *EGR1* expression upon TGF-β1 stimulation fluctuated from medium to medium in our present study. Despite increased TGF-β mRNA and protein expression in parallel with the reduced *PPARG* in all medium formulations, we observed nuclear translocation of EGR1 in only two media. This suggests a culture medium-dependent transcriptional activity for EGR1. In contrast, *EGR2* expression was significantly and similarly induced by TGF-β1 in all investigated media.

Interestingly, the expression pattern of inflammatory mediators IL6 and C3 was also dependent on cell culture media. In the kidney, proximal tubular cells are exposed to various cytokines from glomerular filtration, and they also synthesize complement proteins, including C3 and factors B and H (Gerritsma et al. 1997; Peake et al. 1999). In our study, C3 production seemed to depend on the medium serum content as in DMEM and DMEM/F12-based formulations, and TGF-β-induced *C3* overexpression only in DMEM 5% FBS medium. C3 has been demonstrated to decrease upon TGF-β1 in primary PTEC cells in a serum-free medium supplemented with hormones and EGF (Gerritsma et al. 1998), supporting our observation as *C3* was downregulated by 50% in the serum-free KSFM. The differences in FBS-supplemented media need to be investigated.

### Study limitations

First, it is necessary to consider the shortcomings of cultured HK-2 cells as a model of the proximal tubule. HK-2 cells and many of the available proximal tubule model cell lines fail to replicate the differential expression of apical/basal membrane transporters and metabolizing enzymes. These are one of the characteristics of renal proximal tubular cells in vivo (Sanchez-Romero et al. 2016) with additional display of metabolic zonation with different enzyme machinery and transporters along proximal tubule segments S1, S2 and S3 (Lee et al. 2015), and HK-2 cells might be representative of only one of them. Nevertheless, HK-2 cell line is the most widely used experimental model in translational research (Valdés et al. 2021).

Investigating the long-term effect of TGF-β1-induced gene and protein expression and regulation might provide a more detailed understanding of the effect of different medium formulations in HK-2 cells. We also did not investigate medium additives individually; further studies probably need to investigate the effect on single additives, hormones, growth factors and insulin, e.g., on the TGF-β-induced EMT model in HK-2 cells.

## Conclusions

In conclusion, our study demonstrates how cell culture medium formulation (an essential component for cell growth and morphology) and its modification can affect the HK-2 cell behavior during TGF-β1 induced in in vitro EMT. TGF-β1 stimulates the HK-2 EMT process in any investigated culture medium formulations but with different EGR1 activation and inflammatory response. Choosing cell culture medium formulations and explaining the expression of EMT and pro-fibrotic markers in TGF-β1-induced HK-2 cells is essential and should not be overlooked.

### Supplementary Information

Below is the link to the electronic supplementary material.

Supplementary file1 (PDF 303 KB)

## Data Availability

Experimental data are available upon reasonable request from the corresponding author.

## Abbreviations

ACTA2: Actin alpha 2, smooth muscle
BPE: Bovine pituitary extract
ATCC: American type cell collection
BCA: Bicinchoninic acid
ciPTEC: Human conditionally immortalized proximal tubular epithelial cell line
C3: Complement C3
COL4A1: Collagen type IV alpha 1 chain
CTGF: Connective tissue growth factor
DMEM 2%: Dulbecco’s modified Eagle’s medium supplemented with 2% fetal bovine serum
DAPI: 4′,6-Diamidino-2-phenylindole
DMEM 5%: Dulbecco’s modified Eagle’s medium supplemented with 2% fetal bovine serum
DMEM 10%: Dulbecco’s modified Eagle’s medium supplemented with 2% fetal bovine serum
DMEM/F12 10%: Dulbecco’s Modified Eagle Medium/Nutrient Mixture F-12 supplemented with 10% fetal bovine serum
ECL: Enhanced chemiluminescence
EGR1: Early growth response factor 1
EGR2: Early growth response factor 2
EMT: Epithelial-to-mesenchymal transition
FN: Fibronectin
GAPDH: Glyceraldehyde-3-phosphate dehydrogenase
HK-2: Immortalized proximal tubule epithelial cell line
HPV16: Human papillomavirus
IL6: Interleukin 6
KFSM: Keratinocyte serum-free medium
PTEC: Proximal tubular epithelial cells
PTEC 2%: Hormonally defined medium supplemented with 2% fetal bovine serum
PPARG: Peroxisome proliferator activated receptor gamma
PBS: Phosphate buffer saline
rEGF: Recombinant epidermal growth factor
RPTEC/TERT1: HTERT-immortalized epithelial cell line
SA7K: Pseudo-immortalized human renal proximal tubule epithelial cell line
SD: Standard deviation
TGF-β: Transforming growth factor beta cytokine
TGFB1: Transforming growth factor beta 1
VIM: Vimentin
ZO-1: Zonula occludens-1
