# Supplementary material for: HK-2 cell response to TGF-β highly depends on cell culture medium formulations
Source: Histochem Cell Biol. 2023 Sep 26;161(1):69–79. doi: 10.1007/s00418-023-02237-x (PMC10794419; doi:10.1007/s00418-023-02237-x)
Supplement: Supplementary file 1 — Supplementary file1 (PDF 303 KB) [file 418_2023_2237_MOESM1_ESM.pdf]

Supplemetary file

Title: HK-2 cell response to TGF-β highly depends on cell culture medium formulations.

Authors: Gantsetseg Garmaa[1], Anna Manzéger[1,2], Samaneh Haghighi[1], Gábor Kökény[1,2]*

Affiliations:

[1] Institute of Translational Medicine, Semmelweis University, 1089 Budapest, Nagyvárad tér 4, Hungary

[2] International Nephrology Research and Training Center, Semmelweis University, 1089 Budapest, Nagyvárad tér 4, Hungary

***Corresponding Author**

Gabor Kökény MD, PhD, Dr. habil.,

ORCID: 0000-0002-0345-6914

Email: kokeny.gabor@med.semmelweis-univ.hu, Phone: +361200-200

Semmelweis University, Institute of Translational Medicine, H-1089 Nagyvárad tér 4, Budapest, Hungary

Supplementary Table S1.

**Antibody**	**Manufacturer**
rabbit polyclonal anti-TGF-β1 (3711)	Cell Signaling, Danvers, MA, USA
rabbit polyclonal anti-fibronectin (F3648)	Sigma-Aldrich, St. Louis, MO, USA
rabbit polyclonal anti-EGR2 (NB100-92327)	Novus Biologicals, USA
rabbit mAb anti-E-Cadherin (24E10)	Cell Signaling, Danvers, MA, USA
rabbit mAb anti-Vimentin (D21H3)	Cell Signaling, Danvers, MA, USA
rabbit anti-alfa-SMA (D4k9N)	Cell Signaling, Danvers, MA, USA
mouse mAb anti-Gapdh (MAB374)	Sigma-Aldrich, St. Louis, MO, USA
mouse mAb anti-tubulin	Sigma-Aldrich, St. Louis, MO, USA
HRP-conjugated anti-mouse IgG	Cell Signaling, Danvers, MA, USA
HRP-conjugated anti-rabbit IgG	Cell Signaling, Danvers, MA, USA
Alexa594-conjugated donkey anti-rabbit	Jackson Immunoresearch

Legend. Primary and secondary antibodies used in the study.

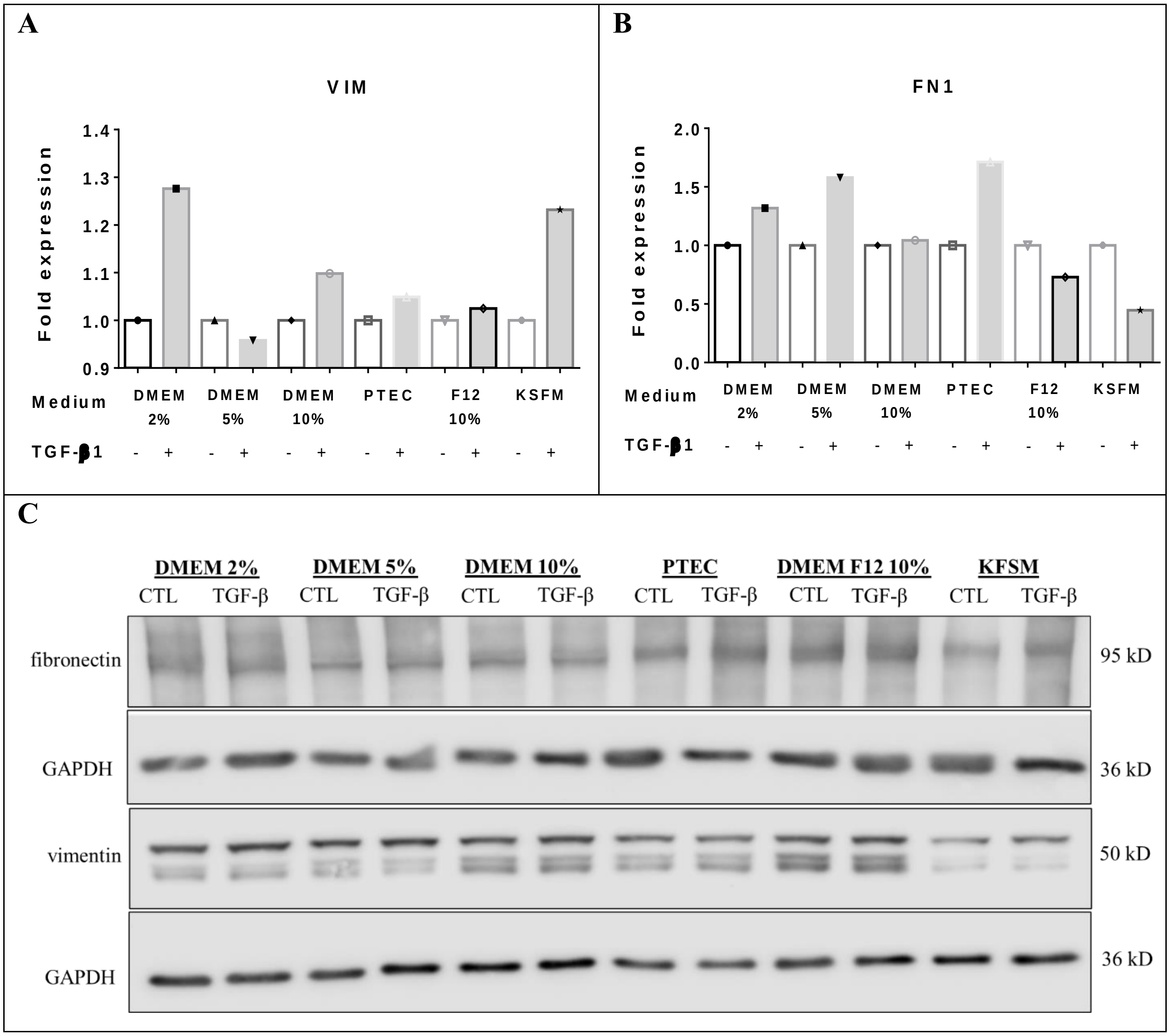

Supplementary Fig. S1. Immunoblots of mesenchymal markers. Immunoblot results of mesenchymal markers (A: vimentin and B: fibronectin) in control (CTL) and TGF-β1 treated (TGFb) HK-2 cells in the six culture medium formulations. GAPDH was used as a loading control. The mean expression for each culture medium group is shown relative to the corresponding controls. C: representative immunoblot. Abbreviations: CTL –control group, TGF-β – transforming growth factor-1 (10 ng/ml) induced group, DMEM 2%; DMEM with 2% FBS supplemented medium, DMEM 5%; DMEM with 10% FBS supplemented medium, DMEM/F12 10%; DMEM with 10% FBS supplemented medium, PTEC; hormonally defined medium, KFSM; Keratinocyte Serum-Free Growth Medium.
